# Validation of the German version of the Mediterranean Diet Adherence Screener (MEDAS) questionnaire

**DOI:** 10.1186/s12885-017-3337-y

**Published:** 2017-05-18

**Authors:** Katrin Hebestreit, Maryam Yahiaoui-Doktor, Christoph Engel, Walter Vetter, Michael Siniatchkin, Nicole Erickson, Martin Halle, Marion Kiechle, Stephan C. Bischoff

**Affiliations:** 1 Institute for Nutritional Medicine, University of Hohenheim, Fruwirthstr. 12, 70593 Stuttgart, Germany; 2 Institute for Medical Informatics, Statistics and Epidemiology, University of Leipzig, Haertelstrasse 16–18, 04107 Leipzig, Germany; 3 Institute for Food Chemistry, University of Hohenheim, Fruwirthstr. 12, 70593 Stuttgart, Germany; 4 Institute for Medical Psychology and Sociology, University Hospital Schleswig-Holstein, Campus Kiel, Preusserstr. 1–9, 24105 Kiel, Germany; 5 Comprehensive Cancer Center (CCC LMU), Ludwig Maximilian University Munich, Campus Großhadern, Marchioninistr. 15, 81377 Munich, Germany; 6 Else Kroener-Fresenius Prevention Center, Klinikum rechts der Isar, Technical University Munich (TUM), Ismaningerstr. 22, 81675 Munich, Germany; 7 Department of Prevention and Sports Medicine, Klinikum rechts der Isar, Technical University Munich (TUM), Ismaningerstr. 22, 81675 Munich, Germany; 8 Department of Gynecology and Center for Hereditary Breast and Ovarian Cancer, Women’s Hospital Klinikum Rechts der Isar der, Technical University Munich (TUM), Gynaecology and Obstetrics, Ismaningerstrasse 22, 81675 Munich, Germany

**Keywords:** Mediterranean diet adherence, Hereditary breast cancer, BRCA1/2, Food frequency, Validation

## Abstract

**Background:**

Health benefits of the Mediterranean Diet (MD) have been shown in different at-risk populations. A German translation of the Mediterranean Diet Adherence Screener (MEDAS) from the PREvención con DIeta MEDiterránea (PREDIMED) consortium was used in the LIBRE study, investigating effects of lifestyle-intervention on women with BRCA1/2 mutations. The purpose of the present study is to validate the MEDAS German version.

**Methods:**

LIBRE is a multicentre (three university hospitals during this pilot phase), unblinded, randomized, controlled clinical trial. Women with a BRCA1/2 mutation of age 18 or over who provided written consent were eligible for the trial. As part of the assessment, all were given a full-length Food Frequency Questionnaire (FFQ) and MEDAS at baseline and after 3 months. Data derived from FFQ was compared to MEDAS in order to evaluate agreement or concordance between the two questionnaires. Additionally, the association of dietary intake biomarkers in the blood (β-carotene, omega-3, omega-6 and omega-9 fatty acids and high-sensitivity C-reactive protein (hsCRP)) with some MEDAS items was analyzed using t-Tests and a multivariate regression.

**Results:**

The participants of the LIBRE pilot study were 68 in total (33 Intervention, 35 Control). Only participants who completed both questionnaires were included in this analysis (baseline: 66, month three: 54). The concordance between these two questionnaires varied between the items (Intraclass correlation coefficient of 0.91 for pulses at the highest and −0.33 for sugar-sweetened drinks). Mean MEDAS scores (sum of all items) were 9% higher than their FFQ counter-parts at baseline and 15% after 3 months. Higher fish consumption (at least 3 portions) was associated with lower omega-6 fatty acid levels (*p* = 0.026) and higher omega-3 fatty acid levels (*p* = 0.037), both results being statistically significant.

**Conclusions:**

We conclude that the German MEDAS in its current version could be a useful tool in clinical trials and in practice to assess adherence to MD.

**Trial registration:**

ClinicalTrials.gov, registered on March 12, 2014, identifier: NCT02087592. World Health Organization Trial Registration, registered on 3 August 2015, identifier: NCT02087592.

## Background

The MD has been tested for its health benefits in different at-risk populations with favourable results. For instance, randomized controlled intervention studies revealed that MD is effective in the primary prevention of cardiovascular diseases [1, 2], in lowering hypertension and atherogenic lipoproteins [3, 4] and in improving diabetes [5]. More recently, studies in the older population showed an association between MD and improved cognitive function [6]. Epidemiological studies from the European Prospective Investigation into Cancer and Nutrition (EPIC) cohort suggested further that MD might protect against cancer, especially gastric cancer [7], colorectal cancer [8] and bladder cancer [9].

Such trials raised the need for a useful tool to assess MD adherence in study populations. The PREDIMED consortium established a 14-point MEDAS questionnaire, which was validated by administering the established FFQ [10] and MEDAS to 7146 participants from the PREDIMED study. The authors found that the average MEDAS score estimate was 105% of the FFQ PREDIMED score estimate, and thus is a valid instrument for rapid estimation of adherence to the MD [11]. Moreover, the PREDIMED investigators could show that MEDAS is able to capture a strong monotonic inverse association between adherence to MD and obesity indices in a population of adults with a high cardiovascular risk [12].

In Germany, a multicentre trial, the ‘Lifestyle intervention in BRCA 1/2-mutation carriers’ (LIBRE) was launched to investigate the effect of a defined lifestyle intervention on breast cancer incidence in women at high genetic risk for this type of cancer [13]. Up to 60% of women with BRCA mutations develop breast or ovarian cancer [14], but not all of them, suggesting that environmental co-factors play a role. Indeed, some studies suggested that physical activity and dietetic intervention help prevent cancer, including breast cancer [15, 16]. To test the hypothesis of whether these controllable environmental factors further modulate cancer risk, the LIBRE trial conducts an intervention with clearly defined sport and nutrition components. The nutritional component of the intervention was based on the MD. The German translation of MEDAS was used as an instrument to assess adherence. The purpose of the present study was to validate the German version of MEDAS.

## Methods

### Study population

The LIBRE study is divided into two parts: firstly, a feasibility study to prove the practicability of the lifestyle intervention and consequently, the presently recruiting main trial with the aim of attaining 600 study participants to demonstrate the effects of the lifestyle intervention on the breast cancer incidence in women with BRCA1 or BRCA2 genetic mutations. 68 women, who were all participants of the LIBRE feasibility study, formed the study population for these analyses. The details of the trial have been published elsewhere [13]. The study population (adult women between 18 and 75 years) was recruited from February 2014 to July 2014 in selected consultation centres of the German Consortium of Hereditary Breast and Ovarian Cancer in Kiel, Cologne and Munich. All participants signed an informed written consent. The trial was approved by the responsible ethical committees.

Study participants were randomized into two groups with a ratio of 1:1, stratified by participating centre and previous breast cancer. The intervention group (IG) (*n* = 35) received a detailed lifestyle intervention over 12 months, and the control group (CG) (*n* = 33) received no intervention, but standard recommendations for a healthier lifestyle. The lifestyle IG received a supervised physical exercise training program and nutritional education based on the MD. In the first 3 months, the nutritional education took place every fortnight, thereafter at monthly intervals. The CG received minimal nutritional education based on the recommendations of the German Society of Nutrition (DGE - https://www.dge.de/en/) “Usual Care in Germany“and some general advice for increasing activity in everyday life at the beginning of the study (one session). All participants were asked to fill out both a full-length FFQ and MEDAS at study start (baseline) and 3 months later. We chose both time points to prove whether MEDAS is specific enough by measuring MD-typical changes during the intervention. Only data collected within this period were used for the purposes of the current study. Solely participants who had completed both questionnaires were included in the analysis. These were in total 66 participants at baseline (34 in the IG and 32 in the CG) and 54 at month three (27 in the IG and 27 in the CG).

We used the guidelines from the International Society for Pharmacoeconomics and Outcomes Research (ISPOR) to guide our translation process [17]. MEDAS from the English PREDIMED-publication [11] was translated into German and reviewed by two native speakers in German. It was then translated back into English by a native speaker. Following this, the final version was read and approved in a small group of the study team.

### Dietary assessment

MEDAS is a 14-item screener, which consists of 12 questions on food consumption frequency and 2 questions on food intake habits characteristic of the MD (Table 1). Each question was scored with a 0 or 1. One point was given for using olive oil as the principal source of fat for cooking and one for preferring white meat over red meat, and one for consuming each of the following:

- 4 or more tablespoons (1 tablespoon = 13.5 g) of olive oil/d (including that used in frying, salads, meals eaten away from home, etc.);
- 2 or more servings of vegetables/d;
- 3 or more pieces of fruit/d;
- fewer than 1 serving of red meat or sausages/d;
- fewer than 1 serving of animal fat/d;
- fewer than 1 cup (1 cup =100 mL) of sugar-sweetened beverages/d;
- 7 or more servings of red wine/wk.;
- 3 or more servings of pulses/wk.;
- 3 or more servings of fish/wk.;
- fewer than 3 commercial pastries/wk.;
- 3 or more servings of nuts/wk.; or
- 2 or more servings/wk. of a dish with a traditional sauce of tomatoes, garlic, onion, or leeks sautéed in olive oil.

**Table 1:** MEDAS questions and transfer of food intake data from FFQ into its food groups

	MEDAS question	data recorded by FFQ
1.	Do you use olive oil as the principal source of fat for cooking?	1 point given: use of olive oil for the preparation of at least 2 of the following groceries: salad, vegetable, meat/fish (FFQ Question: Pages 14 and 19)
2.	How much olive oil do you consume per day (including that used in frying, salads, meals eaten away from home, etc.)?	1 point given: based on FFQ calculation, if >48 g vegetable oil
3.	How many servings of vegetables do you consume per day?	1 point given: based on FFQ calculation, if ≥2 portions of vegetables per day (including salad, olives, mushrooms)
4.	How many pieces of fruit (including fresh-squeezed juice) do you consume per day?	1 point given: based on FFQ calculation, if ≥3 portions of fruit (including mixed fruit, mixed stewed fruit and fruit juices)
5.	How many servings of red meat, hamburger, or sausages do you consume per day?	1 point given: based on FFQ calculation, if <100 g meat (beef, veal, pork, lamb) and processed meat products
6.	How many servings (12 g) of butter, margarine, or cream do you consume per day?	1 point given: based on FFQ calculation, if <1 portion butter, margarine and cream
7.	How many carbonated and/or sugar-sweetened beverages do you consume per day?	1 point given: based on FFQ calculation, sugar-sweetened beverages <1 portion per day (including lemonade and colas)
8.	Do you drink wine? How much do you consume per week?	1 point given: based on FFQ calculation, if ≥7 portions wine (red and white)
9.	How many servings of pulses do you consume per week?	1 point given: ≥ 3 portions pulses per week (page 14)
10.	How many servings of fish/seafood do you consume per week?	1 point given: based on FFQ calculation, if ≥3 portions fish, fish products and seafood per week
11.	How many times do you consume commercial (not homemade) pastry such as cookies or cake per week?	1 point given: based on FFQ calculation, if <3 portions cakes, chocolate, cookies, sweets with and without chocolate per week
12.	How many times do you consume nuts per week?	1 point given: based on FFQ calculation, if ≥3 portions nuts and seeds per week (page 11)
13.	Do you prefer to eat chicken, turkey or rabbit instead of beef, pork, hamburgers, or sausages?	1 point given: based on FFQ calculation, if g white meat (poultry, chicken, rabbit) > g red meat (beef, veal, pork, lamb, processed meat products)
14.	How many times per week do you consume boiled vegetables, pasta, rice, or other dishes with a sauce of tomato, garlic, onion, or leeks sautéed in olive oil?	1 point given: > 1–2 times a week tomato sauce (page 21)

If the condition was not met, 0 points were recorded for the category. The MEDAS score (sum of above items) ranged from 0 to 14 points [11].

All participants were also asked to complete a 148-item semi-quantitative FFQ. The German version had been validated by the German EPIC investigators [18–21]. For each item it questions the average serving size, described by photos, and the food frequency during the previous 12 months. Furthermore, it contains questions on cooking oil, frequency of the use of gravy, the fat content of dairy products, the use of sugar and milk in coffee and tea, and the seasonal consumption of fruit and vegetables. The documentation of the questionnaire was done via *the study-management-system for Epidemiology and Public Health*, which was developed and supervised by the Department of Epidemiology of the German Institute of Human Nutrition Potsdam-Rehbruecke.

Food intake data recorded by FFQ was grouped into the food-based dietary components of MEDAS (Table 1). We validated the dietary assessment data retrieved from MEDAS by comparing it with the data gathered from the validated FFQ and confirmed this by associating with the results from the blood tests.

### Measurement of dietary intake biomarkers in the blood

To confirm whether MEDAS’ tendency towards the MD is consistent, we selected specific biomarkers in the blood which are described in the literature to be associated with consumption of certain MD food components [1, 22, 23].

Blood samples were taken at the same time points as the completion of both questionnaires (baseline and after 3 month). Following 30 to 60 min of incubation the serum was centrifuged at 3000*g for 10 min in the consultation centres and was overnight delivered chilled together with the EDTA (ethylene diamine tetraacetic acid) blood samples to the central laboratory at the University of Hohenheim.

A part of the serum was passed directly to an external laboratory (Medizinisches Labor Bremen, Bremen, Germany) to measure β-carotene by high performance liquid chromatography (HPLC). The rest of the serum aliquots were stored at −80 °C in Hohenheim until the measurement of hsCRP using a sandwich Enzyme Immuno Assay (K 9710S, Immundiagnostik AG, Bensheim, Germany) was done. The erythrocyte membrane was isolated from the EDTA blood and also stored at −80 °C in Hohenheim until the fatty acid profile (omega-6-, omega-3- and omega-9-fatty acids) was analyzed after acid esterification using gas chromatography/mass spectrometry by the Institute of Food Chemistry of the University of Hohenheim [24, 25].

### Statistical analysis

Patient characteristics were analyzed descriptively, split by study arm. For age and Body Mass Index (BMI), a t-Test was used to determine whether the two groups were statistically different. For all other characteristics, coded as binary items, a Chi^2^ test was used.

We then determined the concordance between the answers to the MEDAS questionnaire compared to the answers for corresponding questions in the FFQ questionnaire both at Baseline and at 3 months. First, the absolute agreement in percentage was calculated, which was further investigated using Cohen’s kappa (κ) and the intraclass correlation coefficient (ICC). The relative agreement between the corresponding items was examined using the Pearson product–moment correlation.

The agreement between the sum from the MEDAS questionnaire and the equivalent FFQ questions was examined using a Bland-Altman analysis. The mean of the two values was plotted on the x-axis and the difference on the y-axis, in order to determine possible bias. The 95% limits of agreement lines, defined as the mean difference ± 1.96 times the standard deviation of the differences, were also plotted. A linear regression was then carried out with the difference as the dependent value, whose line was added to the plot with its corresponding formula and *p*-value.

In a further step, we validated whether the MEDAS questionnaire can specifically determine adherence to a MD, the association of blood values for β-carotene, the fatty acids omega-3, omega-6 and omega-9 and hsCRP, and MEDAS items (β-carotene associated with the MEDAS item regarding vegetables and fruits; hsCRP, omega-3 fatty acids, omega-6 fatty acids and omega-9 fatty acids associated with the MEDAS item regarding olive oil; omega-3 fatty acids and omega-6 fatty acids associated with the MEDAS item regarding red meat, fish and nuts). We first applied a t-Test for independent groups (control or intervention) for each of the food items and each of the dietary biomarker values, carried out separately for each of the two time points. Following this, we carried out a multivariate linear regression on the associations described above, where we also adjusted for the study arm.

The statistical analysis was done using R (program for statistical computing) in the R Studio environment Version 0.99.902.

## Results

### Patient characteristics

Patient characteristics at baseline are outlined in Table 2. The IG comprised 35 women at this point in time, while the CG comprised 32. Considering attributes such as BMI, age and history of breast cancer, these groups did not differ statistically significantly from one another. Both groups included a number of smokers (11% in the IG and 9% in the CG). A vegetarian diet was also followed by a group of the study participants (6% in the IG and 13% in the CG).

**Table 2:** Study patient characteristics at baseline (*n* = 67)

	Intervention group (MD) *n* = 34	Control group *N* = 32
Age [years]^a^	42 (27–72)	42 (24–68)
BMI [kg per m^2^]^a^	23 (18–45)	23 (18–43)
History of breast cancer^b^	24 (71%)	21 (66%)
Smoker^b^	4 (11%)	3 (9%)
Vegetarian^b^	2 (6%)	4 (13%)

^a^median (range)

^b^numbers (percent)

### Item by item agreement

The absolute and relative agreements between the FFQ and the MEDAS questionnaires were calculated at baseline and at 3 months for the whole sample (Table 3). This concordance at Baseline was highest for questions 1: olive oil as principal source of fat (Pearson’s product–moment correlation 0.70, κ 0.70 and an ICC of 0.68), and 12: nuts (Pearson’s product–moment correlation 0.72, κ 0.70 and an ICC of 0.68). After 3 months the highest concordance was obtained for questions 9: pulses (Pearson’s product–moment correlation 0.86, κ 0.85 and an ICC of 0.91), and 12: nuts (Pearson’s product–moment correlation 0.78, κ 0.77 and an ICC of 0.76). Questions 7: sugar-sweetened beverages at Baseline and 2: daily olive oil had the lowest concordances with negative values.

**Table 3:** Agreement between MEDAS and FFQ (German)

Question	Baseline (*n* = 66)				3 months (*n* = 54)			
	r	AA	κ	ICC	r	AA	κ	ICC
1	0.70	0.87	0.70 (0.51 to 0.89)	0.68 (0.07 to 1.3)	0.51	0.87	0.51 (0.22 to 0.81)	0.55 (−0.19 to 1.28)
2	0.29	0.86	0.23 (−0.07 to 0.53)	0.21 (−0.35 to 0.76)	−0.09	0.70	−0.034 (−0.1 to 0.03)	−0.03 (−0.08 to 0.02)
3	0.30	0.63	0.28 (0.06 to 0.49)	0.14 (−0.26 to 0.54)	0.25	0.62	0.19(−0.02 to 0.4)	0.15 (−0.36 to 0.66)
4	0.42	0.70	0.36 (0.15 to 0.56)	0.28 (−0.33 to 0.88)	0.08	0.25	0.087 (−0.16 to 0.31)	−0.03 (−0.06 to 0.01)
5	0.58	0.81	0.50 (0.28 to 0.72)	0.52 (−0.20 to 1.24)	0.40	0.87	0.39 (0.03 to 0.75)	0.04 (−0.35 to 1.14)
6	0.45	0.73	0.45 (0.22 to 0.67)	0.32 (−0.33 to 0.97)	0.23	0.61	0.22 (−0.03 to 0.48)	0.06 (−0.20 to 0.33)
7	−0.04	0.91	−0.03 (−0.07 to 0.02)	−0.11 (−0.14 to −0.08)	n/a	0.96	n/a	−0.33 (−0.38 to −0.28)
8	0.35	0.83	0.28 (−0.001 to 0.57)	0.51 (−0.28 to 1.30)	0.24	0.91	0.21 (−0.19 to 0.6)	0.37 (−0.57 to 1.31)
9	n/a	0.92	n/a	n/a	0.86	0.96	0.85 (0.66 to 1.1)	0.91 (0.68 to 1.14)
10	0.25	0.84	0.21 (−0.18 to 0.61)	0.22 (−0.42 to 0.87)	0.28	0.74	0.21 (−0.04 to 0.45)	0.13 (−0.28 to 0.55)
11	0.44	0.77	0.37 (0.14 to 0.6)	0.33 (−0.33 to 0.99)	0.64	0.83	0.61 (0.39 to 0.83)	0.58 (−0.13 to 1.28)
12	0.72	0.86	0.70 (0.52 to 0.87)	0.68 (0.05 to 1.30)	0.78	0.89	0.77 (0.61 to 0.94)	0.76 (0.23 to 1.28)
13	0.18	0.43	0.06 (−0.01 to 0.13)	0.03 (−0.14 to 0.20)	0.13	0.27	0.03 (−0.01 to 0.08)	0.01 (−0.15 to 0.13)
14	0.39	0.69	0.38 (0.16 to 0.6)	0.25 (−0.33 to 0.83)	0.17	0.60	0.17 (−0.01 to 0.43)	0.02 (−0.14 to 0.18)

AA = absolute agreement, r = Pearson’s product–moment correlation, κ = Cohen’s Kappa with the confidence intervals in brackets, ICC = Intraclass Correlation Coefficient with the confidence intervals in brackets

### MEDAS total score agreement

**Fig. 1:**
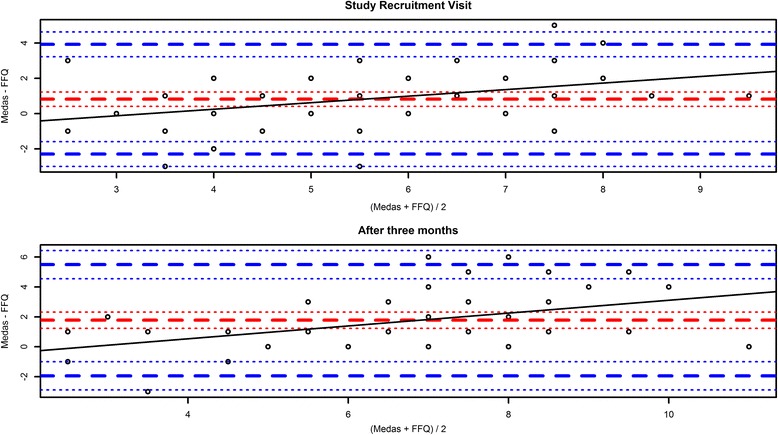
Bland-Altman plots showing the differences between the MEDAS score aggregated from MEDAS and FFQ questionnaires at baseline and after 3 months. The *red dashed* and *dotted lines* indicate the mean bias and its 95% confidence interval. The *blue dashed* and *dotted lines* depict limits of agreement and their 95% confidence intervals

The MEDAS score was analyzed using a Bland-Altman plot (Fig. 1). The mean MEDAS scores were 15% higher than the FFQ score at baseline and 23% higher after 3 months, with the median MEDAS score being higher than the FFQ equivalent by 1 point at baseline and 2 points after 3 months. This corresponds to a mean difference of 1.27 at baseline and 2.12 after 3 months, which defines the bias towards higher score sums being obtained by the MEDAS questionnaire. Linear regression analysis revealed a significant increase of the bias with increasing score values (*p* < 0.001 for baseline, *p* < 0.001 after 3 months).

### Measurement of dietary intake biomarkers in the blood

**Fig. 2:**
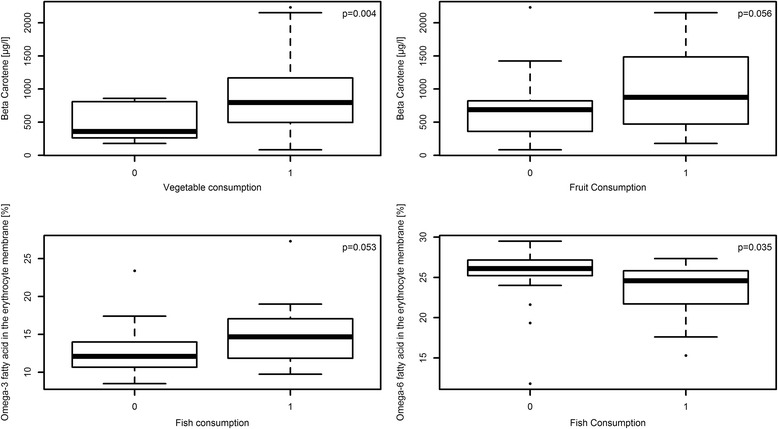
Association of vegetable consumption (question 3 of the MEDAS questionnaire) and fruit consumption (question 4 of the MEDAS questionnaire) with β-carotene; and association of fish consumption (question 10 of the MEDAS questionnaire) with omega-6 fatty acids and omega-3 fatty acids after 3 months (V1) [0 = 0 point in the MEDAS question; 1 = 1 point in the MEDAS question]. The *p*-value was calculated using the t-test

The possible association between laboratory measurements and intake of particular food groups was first analyzed on a per item basis (Table 4). After the 3-month MD intervention some of these associations showed a statistical significance or near-significance. Consumption of at least 3 portions of fish per week was associated with lower omega-6 fatty acid levels (*p* = 0.035) and higher omega-3 fatty acid levels (*p* = 0.053). Consumption of at least 3 portions of fruit per day was associated with higher levels of β-carotene (*p* = 0.056). Consumption of at least 2 portions of vegetables per day was associated with higher levels of β-carotene (*p* = 0.004). We have depicted these associations in Fig. 2 *.*

**Table 4:** MEDAS food groups association with dietary biomarkers in the blood

	Baseline (*n* = 66)			At 3 months(*n* = 54)		
MEDAS food group association with dietary biomarker(s)	Mean for group with 0 point (n)	Mean for group with 1 point (n)	*p*-value	Mean for group with 0 point (n)	Mean for group with 1 point (n)	*p*-value^*^
vegetables with						
β-carotene [μg/l]	859.7 (26)	889.3 (39)	0.868	487.8 (9)	918.9^1^ (45)	**0.004**
fruit with						
β-carotene [μg/l]	786.6 (36)	1009.5^1^ (29)	0.183	689.5 (25)	984.18^1^ (29)	0.056
how much olive oil with						
hsCRP [mg/l]	0.6 (54)	0.5^1^ (11)	0.337	0.7 (39)	0.5^1^ (15)	0.218
omega 3 [%]	14.0 (54)	13.7 (11)	0.763	13.2 (39)	13.8^1^ (15)	0.513
omega 6 [%]	27.5 (54)	27.3 (11)	0. 831	25.0 (39)	25.0^1^ (15)	0.983
omega 9 [%]	14.08 (54)	13.77 (11)	0.185	15.4 (39)	15.16 (15)	0.362
red meat with						
omega 3 [%]	14.2 (21)	13.8 (44)	0.665	12.1 (8)	13.3 (45)	0.202
omega 6 [%]	27.5 (21)	27.4 (44)	0.908	25.0 (8)	25.2 (45)	0.849
fish with						
omega 3 [%]	14.1 (59)	12.6 (6)	0.280	12.6 (38)	15.1^1^ (16)	0.053
omega 6 [%]	27.4 (59)	27.9 (6)	0.629	25.6 (38)	23.4^1^ (16)	**0.035**
nuts with						
omega 3 [%]	13.5 (38)	14.5 (27)	0.230	13.8 (23)	13.2 (30)	0.559
omega 6 [%]	27.8 (38)	26.9 (27)	0.811	24.4 (23)	25.4 (30)	0.361

^*^based on the t-test

Only significant *P* values are bold

We also examined the same associations in a multivariate model, as reported in Table 5. Consumption of at least three portions of fish per week showed increased levels of omega-3 (*p* = 0.037) and decreased levels of omega-6 fatty acid (*p* = 0.026) in the blood, both of which were statistically significant.

**Table 5:** Dietary blood biomarkers association with MEDAS food groups, assessed using multivariate regression

	Baseline		At 3 months	
Dietary biomarker association with MEDAS food group	Estimate	*p*-Value	Estimate	*p*-Value^*^
β-Carotene with				
Fresh vegetables	−22	0.197	446	0.104
Fruits	237	0. 287	366	0.147
omega 3 with				
Amount of olive oil	−0.99	0.415	1.1	0.230
Red meat	−0.99	0.282	2.2	0.099
Fish	−2.1	0.133	2.1	**0.037**
Nuts	1.5	0.094	−1.2	0.225
omega 6 with				
Amount of olive oil	0.269	0.775	−0.4	0.702
Red meat	0.243	0.734	−1.3	0.360
Fish	0.834	0.433	−2.4	**0.026**
Nuts	−0.96	0.162	1.5	0.147
omega 9 with				
Amount of olive oil	−0.095	0.114	−0.054	0.542
HsCRP with				
Amount of olive oil	−0.234	0.402	−0.3	0.161

^*^adjusted for the study arm

Only significant *P* values are bold

## Discussion

MEDAS was developed for the Spanish PREDIMED study to expediently determine adherence to the MD and allow an immediate feedback to the patient. This short screener is a validated tool for the rapid assessment of adherence to the MD [11], which is why it was decided to use a German-translation of this questionnaire in the LIBRE study. However, the original MEDAS was not in German. To validate the German translation of this questionnaire, we used the validated German full-length FFQ as reference.

In general agreement between FFQ and MEDAS questionnaires was of a fair or better level (0.4 and larger values for agreement coefficients) for about half of the MEDAS questions [22]. These differences are likely due to the way FFQ is structured and to the fact that it is differently analyzed. FFQ measures the food frequency of a selected list of German foods with standardized portion sizes for the previous 12 months. These answers are then used to calculate intake for food groups while MEDAS directly asks for the habits and consumption frequency of specific amounts of specific Mediterranean foods during the previous week. In short, the FFQ measures specific details whereas the MEDAS is measuring for components of an overall dietary pattern. For the conversion of food intake data from FFQ into MEDAS food groups only aggregated FFQ data can be used in most cases. For instance, question 3 on vegetables and question 4 on fruits each comprised several questions from FFQ. A further example is that FFQ provides, 3no information on how many times boiled vegetables, pasta, rice, or other dishes with a sauce of tomato, garlic, onion, or leeks sautéed in olive oil are consumed. It asks only about the consumption of tomato sauce, but no further details (e.g. way of cooking or ingredients). Only question 9 (pulses) which has a high concordance is based on a direct answer of FFQ. Additionally, in this study FFQ was completed again within after an interval of just 3 months, which means that the answers for the two time points for FFQ in our analysis overlap for a period of time.

The Bland-Altman analysis showed that the MEDAS score yields higher values for the sum of all items than FFQ with respect to MD. This confirms the results of Schröder et al., who report that the average MEDAS Mediterranean diet score estimate was 105% of the FFQ PREDIMED score estimate [11]. The larger difference between the two score sets after 3 months can be a result of the MD-based intervention in the IG. The MEDAS questionnaire was specifically developed to detect the adherence of an individual’s diet to the principles of the MD. Therefore, MEDAS is more sensitive for MD items than FFQ, resulting in higher scores in individuals abiding by the MD. The FFQ questions, however, assesses the general food intake and does not focus particularly on the consumption of Mediterranean food. Two points can be considered relevant for detecting an adequate implementation of MD in life.

To confirm if the MEDAS’ tendency towards the MD is consistent, we analyzed the association between certain MD food items and selected dietary intake biomarkers in the blood thought to be associated with these food items. The traditional MD is characterized by a high intake of olive oil, fruit, nuts, vegetables, and cereals; a moderate intake of fish and poultry; and a low intake of dairy products, red meat, processed meats, and sweets; while wine is consumed moderately and only together with meals [1, 23]. In the literature, it is described that a high consumption of fruits and vegetables is associated with higher β-carotene blood levels [26]. Kitamura et al. have previously positively correlated frequency of vegetables and fruit intake with β-carotene, among other things [26]. In our study, subjects who consumed at least two portions of vegetables per day according to the MEDAS questionnaire had higher β-carotene blood levels than those who consumed fewer than two portions at both time points. Those who consumed at least three portions of fruit per day according to the MEDAS questionnaire also had higher β-carotene levels in their blood than those who consumed fewer than three portions. This data supports that the MEDAS results reflect the reported intake of particular nutrients charachterisic of a MD pattern.

The MD is rich in poly- and mono-unsaturated fatty acids due to the high consumption of olive oil, fish and nuts [27]. The amount of saturated fatty acids in the MD is lower than in the *Western-style* diet, because red meat and processed meat products play a minor part in the Mediterranean nutrition. The Western-style diet is characterized by its highly processed and refined foods and high contents of sugars, salt and fat and protein from red meat [28]. Olive oil is characterized by a high content of mono-unsaturated fatty acids. Oleic acid (C18:1, n-9) is the main component of olive oil [27]. Therefore, we hypothesized that a high consumption of olive oil, fish and nuts and low red meat intake are associated with changes in the fatty acid profile measured in erythrocyte membrane. Barcelo et al. described elevated values of omega-3-fatty acids and low values of omega-6-fatty acids following high olive oil consumption, while the omega-9-fatty acid amount remained unchanged [29]. Our data demonstrated that, more than four tablespoons of olive oil per day were associated with a tendency to higher serum levels of all unsaturated fatty acids (omega-6, −3 and −9) compared with the values measured in subjects who consumed less olive oil. Takkumen et al. described an association between high fish consumption and a change in the omega-6 and −3-fatty acids profile. The amount of omega-6-fatty acids decreased while that of omega-3-fatty acids increased [30]. At least three portions of fish and seafood per week were statistically significantly associated with lower omega-6-fatty acids values (24% compared to 26.3%, *p* = 0.016) and higher omega-3-fatty acids values. While high meat consumption is associated with higher omega-6-fatty acids values [30], such tendencies could also be seen in this study.

Barceló et al. [29] also reported an association between hsCRP values and olive oil consumption. According to their data, a MD enriched with olive oil (1 litre per week) resulted in a reduction of the plasma hsCRP concentration. Such tendencies could also be seen in this study. Individuals who consume more than four tablespoons of olive oil per day had lower values of hsCRP than individuals who consume less olive oil. The described associations between certain food items and blood values indicate that the MEDAS score indeed reflects a MD. Within this context, MEDAS provides reasonable estimates to adequately rank MD adherence.

Study limitations comprise firstly, a small sample size meaning the statistical tests would only have small power. A further limitation of this study is that our findings may not apply to the general population as the participants belonged to a selected population who may have a particular dietary behaviour due to their knowledge about their genetic disposition for breast cancer.

We will be using the German MEDAS in our main trial that aims to recruit 600 study participants. We decided to use the adherence to MD measured by the MEDAS score as one of 3 co-primary endpoints [31].

## Conclusions

Despite the study limitations, we conclude that the present version of MEDAS could be a reasonable tool in determining adherence to a MD in German-speaking populations. This short screener is a valid tool for the rapid assessment of adherence to the MD that may also be useful not only for trials but also in clinical practice. The MEDAS score would allow an immediate feedback to study participants or patients regarding their adherence to MD.

## Abbreviations

BMI: Body Mass Index
EPIC: European Prospective Investigation into Cancer and Nutrition
EDTA: ethylene diamine tetraacetic acid
FFQ: Food Frequency Questionnaire
HPLC: high performance liquid chromatography
hsCRP: High-sensitivity C-reactive protein
ICC: Intraclass correlation
ISPOR: International Society For Pharmacoeconomics and Outcomes Research
K: Cohen’s Kappa
MEDAS: Mediterranean Diet Adherence Screener
MD: Mediterranean Diet
LIBRE: Lifestyle intervention in BRCA 1/2-mutation carriers
PREDIMED: PREvención con DIeta MEDiterránea (PREvention with MEDiterranean Diet)

## References

[1] Estruch R, Ros E, Salas-Salvado J, Covas MI, Corella D, Aros F, Gomez-Gracia E, Ruiz-Gutierrez V, Fiol M, Lapetra J (2013). Primary prevention of cardiovascular disease with a Mediterranean diet. N Engl J Med.

[2] Ruiz-Canela M, Estruch R, Corella D, Salas-Salvado J, Martinez-Gonzalez MA (2014). Association of Mediterranean diet with peripheral artery disease: the PREDIMED randomized trial. JAMA.

[3] Damasceno NR, Sala-Vila A, Cofan M, Perez-Heras AM, Fito M, Ruiz-Gutierrez V, Martinez-Gonzalez MA, Corella D, Aros F, Estruch R (2013). Mediterranean diet supplemented with nuts reduces waist circumference and shifts lipoprotein subfractions to a less atherogenic pattern in subjects at high cardiovascular risk. Atherosclerosis.

[4] Domenech M, Roman P, Lapetra J, de la Corte FJ G, Sala-Vila A, de la Torre R, Corella D, Salas-Salvado J, Ruiz-Gutierrez V, Lamuela-Raventos RM (2014). Mediterranean diet reduces 24-hour ambulatory blood pressure, blood glucose, and lipids: one-year randomized, clinical trial. Hypertension.

[5] Esposito K, Maiorino MI, Petrizzo M, Bellastella G, Giugliano D (2014). The effects of a Mediterranean diet on the need for diabetes drugs and remission of newly diagnosed type 2 diabetes: follow-up of a randomized trial. Diabetes Care.

[6] Valls-Pedret C, Sala-Vila A, Serra-Mir M, Corella D, de la Torre R, Martinez-Gonzalez MA, Martinez-Lapiscina EH, Fito M, Perez-Heras A, Salas-Salvado J (2015). Mediterranean diet and age-related cognitive decline: a randomized clinical trial. JAMA Intern Med.

[7] Buckland G, Agudo A, Lujan L, Jakszyn P, Bueno-de-Mesquita HB, Palli D, Boeing H, Carneiro F, Krogh V, Sacerdote C (2010). Adherence to a Mediterranean diet and risk of gastric adenocarcinoma within the European prospective Investigation into cancer and nutrition (EPIC) cohort study. Am J Clin Nutr.

[8] Bamia C, Lagiou P, Buckland G, Grioni S, Agnoli C, Taylor AJ, Dahm CC, Overvad K, Olsen A, Tjonneland A (2013). Mediterranean diet and colorectal cancer risk: results from a European cohort. Eur J Epidemiol.

[9] Buckland G, Ros MM, Roswall N, Bueno-de-Mesquita HB, Travier N, Tjonneland A, Kiemeney LA, Sacerdote C, Tumino R, Ljungberg B (2014). Adherence to the Mediterranean diet and risk of bladder cancer in the EPIC cohort study. Int J Cancer.

[10] Fernandez-Ballart JD, Pinol JL, Zazpe I, Corella D, Carrasco P, Toledo E, Perez-Bauer M, Martinez-Gonzalez MA, Salas-Salvado J, Martin-Moreno JM (2010). Relative validity of a semi-quantitative food-frequency questionnaire in an elderly Mediterranean population of Spain. Br J Nutr.

[11] Schröder H, Fito M, Estruch R, Martinez-Gonzalez MA, Corella D, Salas-Salvado J, Lamuela-Raventos R, Ros E, Salaverria I, Fiol M (2011). A short screener is valid for assessing Mediterranean diet adherence among older spanish men and women. J Nutr.

[12] Martinez-Gonzalez MA, Garcia-Arellano A, Toledo E, Salas-Salvado J, Buil-Cosiales P, Corella D, Covas MI, Schroder H, Aros F, Gomez-Gracia E (2012). A 14-item Mediterranean diet assessment tool and obesity indexes among high-risk subjects: the PREDIMED trial. PLoS One.

[13] Kiechle M, Engel C, Berling A, Hebestreit K, Bischoff S, Dukatz R, Gerber WD, Siniatchkin M, Pfeifer K, Grill S (2016). Lifestyle intervention in BRCA1/2 mutation carriers: study protocol for a prospective, randomized, controlled clinical feasibility trial (LIBRE-1 study). Pilot Feasibility Stud.

[14] Mavaddat N, Peock S, Frost D, Ellis S, Platte R, Fineberg E, Evans DG, Izatt L, Eeles RA, Adlard J (2013). Cancer risks for BRCA1 and BRCA2 mutation carriers: results from prospective analysis of EMBRACE. J Natl Cancer Inst.

[15] Friedenreich CM (2010). The role of physical activity in breast cancer etiology. Semin Oncol.

[16] King MC, Marks JH, Mandell JB (2003). Breast and ovarian cancer risks due to inherited mutations in BRCA1 and BRCA2. Science.

[17] Wild D, Grove A, Martin M, Eremenco S, McElroy S, Verjee-Lorenz A, Erikson P (2005). Principles of good practice for the translation and cultural adaptation process for Patient-Reported Outcomes (PRO) measures: report of the ISPOR task force for translation and cultural adaptation. Value Health.

[18] Kroke A, Klipstein-Grobusch K, Voss S, Möseneder J, Thielecke F, Noack R, Boeing H (1999). Validation of a self-administered food-frequency questionnaire administered in the European Prospective Investigation into Cancer and nutrition (EPIC) study: comparison of energy, protein, and macronutrient intakes estimated with the doubly labeled water, urinary nitrogen, and repeated 24-h dietary recall methods. Am J Clin Nutr.

[19] Boeing H, Bohlscheid-Thomas S, Voss S, Schneeweiss S, Wahrendorf J (1997). The relative validity of vitamin intakes derived from a food frequency questionnaire compared to 24-hour recalls and biological measurements: results from the EPIC pilot study in Germany. European prospective Investigation into cancer and nutrition. Int J Epidemiol.

[20] Bohlscheid-Thomas S, Hoting I, Boeing H, Wahrendorf J (1997). Reproducibility and relative validity of food group intake in a food frequency questionnaire developed for the German part of the EPIC project. European prospective Investigation into cancer and nutrition. Int J Epidemiol.

[21] Bohlscheid-Thomas S, Hoting I, Boeing H, Wahrendorf J (1997). Reproducibility and relative validity of energy and macronutrient intake of a food frequency questionnaire developed for the German part of the EPIC project. European prospective Investigation into cancer and nutrition. Int J Epidemiol.

[22] Cicchetti DV (1994). Guidelines, criteria, and rules of thumb for evaluating normed and standardized assessment instruments in psychology. Psychol Assess.

[23] Willett W, Sacks F, Trichopoulou A, Drescher G, Ferro-Luzzi A, Helsing E, Trichopoulos D (1995). Mediterranean diet pyramid: a cultural model for healthy eating. Am J Clin Nutr.

[24] Thurnhofer S, Lehnert K, Vetter W (2007). Exclusive quantification of methyl-branched fatty acids and minor 18:1-isomers in foodstuff by GC/MS in the SIM mode using 10, 11-dichloroundecanoic acid and fatty acid ethyl esters as internal standards. Eur Food Res Technol.

[25] Thurnhofer S, Vetter W (2006). Application of ethyl esters and d3-methyl esters as internal standards for the gas chromatographic quantification of transesterified fatty acid methyl esters in food. J Agric Food Chem.

[26] Kitamura Y, Tanaka K, Kiyohara C, Hirohata T, Tomita Y, Ishibashi M, Kido K (1997). Relationship of alcohol use, physical activity and dietary habits with serum carotenoids, retinol and alpha-tocopherol among male Japanese smokers. Int J Epidemiol.

[27] Wahrburg U, Kratz M, Cullen P (2002). Mediterranean diet, olive oil and health. Eur J Lipid Sci Technol.

[28] Odermatt A (2011). The Western-style diet: a major risk factor for impaired kidney function and chronic kidney disease. Am J Physiol Ren Physiol.

[29] Barcelo F, Perona JS, Prades J, Funari SS, Gomez-Gracia E, Conde M, Estruch R, Ruiz-Gutierrez V (2009). Mediterranean-style diet effect on the structural properties of the erythrocyte cell membrane of hypertensive patients: the Prevencion con Dieta mediterranea study. Hypertension.

[30] Takkunen M, Agren J, Kuusisto J, Laakso M, Uusitupa M, Schwab U (2013). Dietary fat in relation to erythrocyte fatty acid composition in men. Lipids.

[31] Kiechle M, Engel C, Berling A, Hebestreit K, Bischoff SC, Dukatz R, Siniatchkin M, Pfeifer K, Grill S, Yahiaoui-Doktor M (2016). Effects of lifestyle intervention in BRCA1/2 mutation carriers on nutrition, BMI, and physical fitness (LIBRE study): study protocol for a randomized controlled trial. Trials.

